# Support for pictorial health warning labels on cigarette packages in the United States among adults who currently smoke or quit smoking: Findings from the ITC US Smoking and Vaping Surveys

**DOI:** 10.18332/tid/166001

**Published:** 2023-06-23

**Authors:** Shannon Gravely, Gang Meng, David Hammond, Pete Driezen, James F. Thrasher, Geoffrey T. Fong, Lorraine V. Craig, Janet Chung-Hall, Anne C. K. Quah, Janine Ouimet, Maansi Bansal-Travers, K. Michael Cummings

**Affiliations:** 1 Department of Psychology, University of Waterloo, Waterloo, Canada; 2 School of Public Health Sciences, University of Waterloo, Waterloo, Canada; 3 Department of Health Promotion, Education, and Behavior, Arnold School of Public Health, University of South Carolina, Columbia, United States; 4 Ontario Institute for Cancer Research, Toronto, Canada; 5 Department of Health Behavior, Roswell Park Comprehensive Cancer Center, Buffalo, United States; 6 Department of Psychiatry and Behavioral Sciences, Medical University of South Carolina, Charleston, United States

**Keywords:** cigarettes, tobacco, health warning labels, support

## Abstract

**INTRODUCTION:**

In March 2020, the US Food and Drug Administration (FDA) finalized new pictorial health warnings (PHWs), covering 50% of the front and back of the pack; however, legal challenges from cigarette manufacturers have prevented the new warnings from being implemented. About 70% of adults in the general US population support PHWs. This study assessed support for PHWs in 2016, 2018 and 2020 among US adults (aged ≥18 years) who currently smoke or formerly smoked cigarettes. We also assessed factors related to support.

**METHODS:**

Respondents included adults who currently or formerly smoked cigarettes and participated in at least one wave of the US ITC Smoking and Vaping Surveys: Wave 1 (2016, n=2557); Wave 2 (2018, n=2685); and Wave 3 (2020, n=1112). We assessed changes in support for PHWs between 2016 and 2020, and assessed factors related to support (support vs oppose/don’t know). Analyses were conducted on weighted data.

**RESULTS:**

Overall, 38.0% of respondents supported PHWs in 2016, with a significant increase to 44.7% in 2018 (p<0.001), and leveling off to 45.0% in 2020 (2018 vs 2020, p=0.91). Support was highest among former smokers and lowest among daily smokers in all three survey years. Support for PHWs at all survey years was significantly higher among those who formerly smoked, were younger (aged 18–39 vs ≥40 years), those who identified as Black (vs White), and planned to quit smoking (vs not planning to quit). There were no differences by income level, education level, or sex.

**CONCLUSIONS:**

Nearly half of US adults who smoke cigarettes or quit smoking supported PHWs in 2020, with support being higher among younger adults, ethnic minorities, and those who formerly smoked. Support increased between 2016 and 2018, but not between 2018 and 2020. Similar to other studies, fewer current and former smokers supported PHWs compared to the US adult general population.

## INTRODUCTION

In 2009, the US Food and Drug Administration (FDA) was granted authority to regulate tobacco products, including mandating pictorial health warnings (PHWs) covering 50% of the front and back of the principal display areas of cigarette packs. In June 2011, FDA proposed nine (graphic) PHWs that must occupy the top 50% of the front and back of cigarette packages. Cigarette companies challenged the PHWs as violating their First Amendment rights, and, in 2012, the US Court of Appeals struck down the proposed PHWs. In March 2020, the FDA finalized the ‘Required warnings for cigarette packages and advertisements’ rule, establishing 11 new PHWs (Supplementary file Figure 1) that may better survive constitutional challenges^1,2^. Undeterred, cigarette manufacturers have continued litigation to postpone the effective date of the rule; the most recent postponement being granted in December 2022^1-3^.

Evaluating support for tobacco control policies can build the case for policy implementation and serve as a measurement of a policy’s success. In particular, a key factor in creating political will is the level of public support, particularly among those most affected by policy implementation – as is the case for support for tobacco control policies among people who smoke. Previous studies have found majority support for PHWs among the US adult general population (about 70%), with lower support among adults who smoke^4,5^. A previous published study that used data from the 2016 (Wave 1) International Tobacco Control Smoking and Vaping (ITC 4CV) Survey and assessed support for various tobacco control policies, including PHWs, found that 40% of US adults who smoke supported them^6^. The current study updates and extends the previous ITC 4CV study^6^, and assesses changes in support for PHWs between 2016, 2018, and 2020 among US adults who currently smoke or formerly smoked cigarettes. We also assess factors related to support.

## METHODS

### Study design, setting, and participants

The current study used data from Wave 1 (July–September 2016, n=2557), Wave 2 (February–July 2018, n=2685) and Wave 3 (February–June 2020, n=1112) of the US ITC 4CV Surveys, and included US adults (aged ≥18 years) who smoked cigarettes (≥ monthly, n=4392) or quit smoking (n=783).

US respondents were recruited from the Ipsos online probability-based panel, which is nationally representative of the US population across age, sex, geographical region, and socioeconomic status. All panelists who submitted a valid survey in Wave 1 were eligible for Wave 2, and those who completed Wave 2 were invited back to complete Wave 3. Respondents lost to follow-up were replaced at each wave by eligible new panelists using the same sampling procedures. Retention for the US samples were 43% between Waves 1 and 2, and 48% between Waves 2 and 3. Respondents herein completed at least one survey, and provided informed consent electronically before proceeding to take the survey.

### Data weighting

Sampling weights adjust for the oversampling of some sub-populations, non-response, and other sources of bias^7^. Survey weights were designed to make the sample as representative as possible of the US adult general population who smoke or formerly smoked, with respect to sex, age group, education level, and geographical region. National Health Interview Surveys (NHIS) were used as the benchmark to compute weights for US data. Further details about the study procedures, sampling, and data weighting can be found elsewhere^8-10^.

### Measures

The US ITC 4CV surveys are available at: https://itcproject.org/surveys/united-states-america/

*Outcome variable*

Respondents were asked: ‘Would you support or oppose a law that places pictorial health warnings on the front of all cigarette and roll-your-own tobacco packs?’. Response options were ‘strongly support’, ‘support’, ‘oppose’, ‘strongly oppose’, or ‘I don't know’. In 2016 and 2018, all respondents received this survey question; however, in 2020, half of respondents were randomly assigned to receive a set of questions about cigarette regulation. Initially, this outcome measure included all response options to assess the level of support for PHWs at each survey. To assess changes in support between 2016 and 2020, and factors related to support, a dichotomous variable was created by grouping together the options: ‘strongly support’ and ‘support’ as ‘support’; and the options ‘strongly oppose’, ‘oppose’, and ‘don’t know’ as ‘no support’.

*Independent variables*

Sociodemographic data were collected by Ipsos and verified by the respondents at the time of survey completion. Sociodemographic variables were: age group (18–24, 25–39, 40–54, and ≥55 years), sex (male, female), race/ethnicity (White, Black, Hispanic/Latino, other), income level (low, moderate, high, or not reported), and education level (low, moderate, high). Smoking behaviors were: smoking status (daily, non-daily, quit smoking), and plans to quit smoking (yes intending to quit smoking, not intending to quit/don’t know, not applicable, already quit smoking).

### Statistical analysis

Unweighted descriptive statistics were used to describe the study sample (Supplementary file Table 1). All other analyses were conducted on weighted data. Analyses were conducted using SAS-callable SUDAAN (V.11.0.3, RTI International) to account for the stratified sampling design and sampling weights. Statistical significance and confidence intervals were computed at the 95% confidence level.

**Figure 1:**
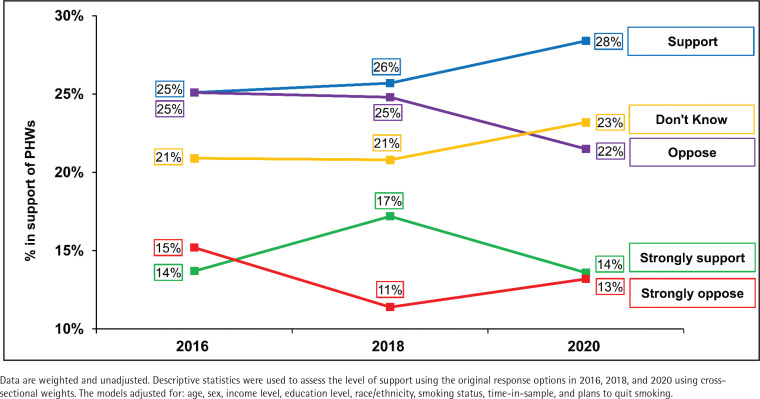
Support for pictorial health warnings in 2016 (N=2557), 2018 (N=2685) and 2020 (N=1112) among adults who currently smoke or quit smoking in the United States

**Figure 2:**
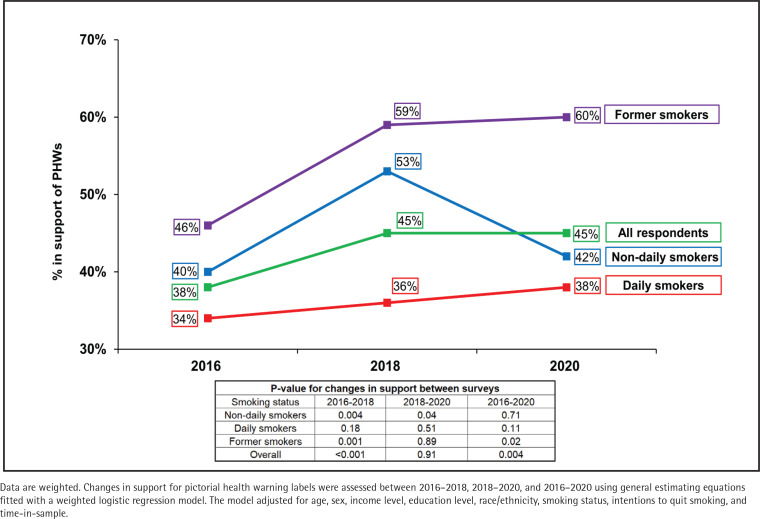
Trends in support for pictorial health warnings on cigarette packs among adults who currently or formerly smoked in the United States, 2016–2020 (N=5175)

In the first analysis, descriptive statistics were computed on (unadjusted) weighted data to assess the level of support using the original response options (‘strongly support’, ‘support’, ‘oppose’, ‘strongly oppose’, ‘I don’t know’) in 2016, 2018, and 2020 using cross-sectional weights (Figure 1 and Supplementary file Figure 2). Secondly, adjusted logistic regression models were conducted on weighted data for each survey year to identify factors associated with support for PHWs (Table 1, Models 1–3). The three models included sex, age group, ethnicity, income level, education level, smoking status, plans to quit smoking, and ‘time-in-sample’ (the number of waves that the respondent had completed). The outcome was dichotomized as ‘support’ (strongly support/support) vs ‘no support’ (strongly oppose/oppose/don’t know). Third, changes in support for PHWs were assessed between 2016–2018, 2018–2020, and 2016–2020 using general estimating equations fitted with a weighted and adjusted logistic regression model (Figure 2). The Zeger method was used for estimating standard errors of regression coefficients to account for the clustered design. The results are presented overall and by smoking status.

**Table 1:** Adjusted logistic regression models assessing factors associated with support for pictorial health warnings on cigarette packs among adult current and former smokers in the United States

*Characteristics*	*Model 1: Wave 1 (2016) (N=2557)*		*Model 2: Wave 2 (2018) (N=2685)*		*Model 3: Wave 3 (2020) (N=1112)*	
	*Weighted %*	*AOR (95% CI)*	*Weighted %*	*AOR (95% CI)*	*Weighted %*	*AOR (95% CI)*
**Sex**						
Male	38.5	1.01 (0.77–1.32)	42.3	1.02 (0.81–1.29)	42.0	1.01 (0.71–1.44)
Female (Ref.)	39.1	1	43.7	1	42.1	1
**Age** (years)						
18–24 (Ref.)	56.7	1	59.8	1	67.4	1
25–39	44.7	0.79 (0.47–1.34)	54.3	0.75 (0.49–1.14)	52.5	0.62 (0.33–1.17)
40–54	32.8	0.52 (0.30–0.88)*	36.7	0.40 (0.26–0.60)*	36.7	0.35 (0.19–0.64)*
≥55	32.3	0.57 (0.34–0.95)*	31.2	0.32 (0.21–0.47)*	31.2	0.26 (0.15–0.48)*
**Smoking status**						
Daily	33.5	0.30 (0.18–0.50)*	33.7	0.40 (0.26–0.61)*	36.2	**0.36 (0.18–0.75)**
Non-daily	47.0	0.41 (0.22–0.75)*	54.7	0.85 (0.51–1.43)	44.9	**0.40 (0.17–0.92)**
Quit (Ref.)	47.2	1	57.8	1	55.5	1
**Income level** ^a^						
Low	36.1	0.82 (0.59–1.16)	43.6	1.07 (0.81–1.42)	41.9	1.05 (0.68–1.62)
Moderate	38.9	0.93 (0.66–1.31)	43.3	1.01 (0.76–1.35)	43.2	1.13 (0.71–1.78)
High (Ref.)	41.0	1	42.4	1	41.4	1
**Education level** ^b^						
Low	39.0	1.04 (0.71–1.52)	43.8	1.39 (1.01–1.92)^†^	43.2	1.03 (0.64–1.67)
Moderate	35.9	0.76 (0.53–1.08)	42.1	1.07 (0.79–1.45)	38.9	0.78 (0.49–1.23)
High (Ref.)	44.4	1	42.3	1	45.7	1
**Race/ethnicity**						
White (Ref.)	36.2	1	41.0	1	39.6	1
Black	46.4	1.70 (1.03–2.81)*	55.6	1.81 (1.16–2.81)*	51.8	1.76 (1.06–2.95)*
Hispanic/Latino	56.1	1.98 (1.21–3.26)*	51.9	1.26 (0.82–1.95)	42.0	0.92 (0.46–1.83)
Other	38.0	1.03 (0.60–1.76)	40.1	0.83 (0.47–1.44)	43.9	1.17 (0.46–2.98)
**Plans to quit smoking**						
Not applicable (quit smoking)	40.9	0.88 (0.51–1.51)	52.7	2.13 (1.34–3.39)*	48.7	1.67 (0.77–3.61)
Planning to quit	42.3	2.17 (1.50–3.13)*	42.3	2.44 (1.73–3.44)*	22.4	2.78 (1.60–4.80)*
Not planning to quit (Ref.)	23.5	1	21.4	1	45.0	1
**Time-in-sample**						
Completed one survey	50.8	1.61 (1.14–2.27)*	46.2	0.87 (0.63–1.18)	42.8	0.97 (0.61–1.56)
Completed two surveys	36.5	1.05 (0.74–1.48)	41.3	0.91 (0.69–1.20)	44.1	0.91 (0.58–1.43)
Completed more than 2 surveys (Ref.)	35.2	1	42.2	1	39.1	1

Data are weighted and adjusted. Outcome (adjusted logistic regressions): support vs no support (oppose/don’t know). Weighted % refers to respondents who support pictorial health warnings. AOR: adjusted odds ratio.

* Statistically significant at p<0.05.

† Main omnibus test was not significant (p=0.07).

a Annual household income is defined as: low (<US$ 30000), moderate (US$ 30000–59000) or high (≥US$ 60000). Respondents (n=26) who did not report their income were excluded.

b Education is defined as: low (≤ high school), moderate (trade school, community college, associate degree, or some university - no degree) or high (university degree or postgraduate degree).

## RESULTS

Characteristics of the study sample at recruitment are presented in Supplementary file Table 1. Unweighted data were used to describe the sample included in this study, whereas all subsequent analyses were conducted on weighted data to describe the US population of adults who smoke or quit smoking.

Table 1 presents the adjusted logistic regression models assessing factors associated with support for PHWs. Overall, sex, income level, and education level were not associated with differences in support in any of the survey years. Younger respondents (aged 18–39 years) were significantly more likely to support PHWs compared to older respondents (aged ≥40 years), and those who identified as White were less likely to support PHWs labels compared to those who identified as Black in all survey years. Those who identified as Hispanic/Latino had significantly higher support than White people in 2016; their rates of support remained higher in 2018 and 2020, but were no longer statically significant. In all survey years, people who were smoking daily were less likely to support PHWs compared to people who had quit smoking. Those smoking non-daily were less likely to support PHWs in 2016 and 2018 compared to those who quit smoking, but there was no difference between the two groups in 2018. Those who were planning to quit smoking were more likely to support PHWs compared to those who were not.

Figure 1 presents the level of support at each survey year. Figure 2 presents the findings for changes in support between 2016 and 2020, overall and by smoking status (Supplementary file Table 2 provides sample sizes and 95% confidence intervals). Support for PHWs increased between 2016 and 2018 (p<0.001), and between 2016 and 2020 (p=0.004), but did not change between 2018 and 2020 (p=0.91). Support varied by smoking status with daily smokers least supportive and former smokers most supportive. Between 2016 and 2018, support increased among non-daily smokers (p=0.004) and former smokers (p=0.001). Between 2018 and 2020, support decreased among non-daily smokers (p=0.04) and increased again among former smokers (p=0.02). Support did not change between any time points for daily smokers (all p≥0.05).

## DISCUSSION

In 2020, nearly half of US adults who smoked or formerly smoked supported PHWs. Support varied by smoking status, with former smokers far more supportive than current smokers. Daily smokers were less supportive than non-daily smokers, although support was higher among current smokers reporting greater interest in quitting smoking (relative to those not intending to quit). Support was also higher among younger age groups and those who identified as Black (compared to White). Our finding that the highest level of support was among those who were aged 18–24 years is encouraging considering that they represent those who would be the ‘next generation’ of adults who would smoke and subsequently suffer from the burden of tobacco use.

The level of support for PHWs observed in our study was lower than previous studies that assessed support among the US adult general population. In 2020, a nationally representative cross-sectional study by Kaufman et al.^4^ found that 70% of US adults supported PHWs; however, they also found that current smokers had almost twice the odds of being neutral or opposed to PHWs than non-smokers. A study by Kamyab et al.^5^ found that support increased among the US adult general population between 2007 (58%) and 2009 (74%) and among non-smokers (65–78%), after which support remained stable until 2012. Among current smokers, support increased between 2007 (35%) and 2011 (62%), but then decreased to 40% in 2012. The study did not assess support among former smokers specifically.

Our study found an increase in support for PHWs between 2016 and 2018 (38–45%), and then remained stable thereafter (45% in 2020), which was similar to previous trends found by Kamyab et al.^5^ (e.g. increased and then plateaued). In contrast, we found that support remained stable across time for daily smokers, whereas there was a significant increase among non-daily smokers between 2016 and 2018, and then a significant decrease between 2018 and 2020, although this may reflect that the studies reported trends over different time periods without any overlapping time period with our study. Notably, non-daily smoking has increased across time^11,12^. Similar to Kamyab et al.^13-15^, we also found that support was higher among people who smoked less, those who had an interest in quitting smoking, former smokers, and younger people. Those who identified as Hispanic or Black reported higher levels of support than those who identified as White. The similarities between this study and our study suggests that these sub-groups have remained stable in their support across time.

### Limitations

Our study is subject to some limitations. First, respondents considered hypothetical PHWs without reference to specific PHWs. Support may vary depending on the content and graphic nature of the warnings. Second, this sample was representative of adults who smoked or formerly smoked, but not of the US general population. Finally, although the weights for our data were designed to make the sample as representative as possible of the US smoking population, the decision to participate in an online panel is likely to depend on some unmeasured characteristics that may be associated with outcomes or relationships of interest. The NHIS survey used for calibrating the survey weights also has limitations, thus the findings in this study should be interpreted with some caution.

## CONCLUSIONS

Pictorial health warnings on cigarette packs are a cost-effective policy to promote awareness of smoking-related harms and smoking cessation. However, in the US, where text warnings have been in place on the side of the pack since 1985, few smokers pay attention to them^16,17^, and the tobacco industry continues to use the entire front and back of the pack for brand marketing. Mandating large PHWs on the principal display of packs is a critical part of a comprehensive tobacco control strategy^15^, and should be implemented without further delay in order to promote smoking cessation and continued abstinence among those who have quit, as well as deter young people from initiating smoking.

## Data Availability

In each country participating in the International Tobacco Control Policy Evaluation (ITC) Project, the data are jointly owned by the lead researcher(s) in that country and the ITC Project at the University of Waterloo. Data from the ITC Project are available to approved researchers two years after the date of issuance of cleaned data sets by the ITC Data Management Centre. Researchers interested in using ITC data are required to apply for approval by submitting an International Tobacco Control Data Repository (ITCDR) request application and subsequently to sign an ITCDR Data Usage Agreement. The criteria for data usage approval and the contents of the Data Usage Agreement are described online (http://www.itcproject.org).
