# Supplementary material for: Support for pictorial health warning labels on cigarette packages in the United States among adults who currently smoke or quit smoking: Findings from the ITC US Smoking and Vaping Surveys
Source: Tob Induc Dis. 2023 Jun 23;21:84. doi: 10.18332/tid/166001 (PMC10288539; doi:10.18332/tid/166001)
Supplement: Supplementary file 1 [file TID-21-84-s1.pdf]

Support for pictorial health warning labels on cigarette packages in the United States among adults who currently smoke or quit smoking: Findings from the ITC US Smoking and Vaping Surveys

Supplemental Table 1. Characteristics of the study respondents at recruitment (unweighted): US International Tobacco Control Smoking and Vaping Surveys, Waves 1 (July-September 2016, n=2,557), 2 (February-July 2018, n=2,685) and 3 (February-June 2020, n=1,112).

Characteristic		N=5,175	Unweighted %
**Sex**	Male	2,526	48.8
	Female	2,649	51.2
**Age group**	18-24	1,456	28.1
	25-39	1,105	21.4
	40-54	916	17.7
	55 and up	1,698	32.8
**Annual household income**	Low	1,765	34.1
	Medium	1,486	28.7
	High	1,898	36.7
	Not Stated	26	0.5
**Highest level of education**	Low	1,780	34.4
	Medium	2,063	39.9
	High	1,331	25.7
**Race/ethnicity**	White	3,819	73.8
	Black	455	8.8
	Hispanic/Latino	558	10.8
	Other	343	6.6
**Smoking status at recruitment**	Daily smoker	3,522	68.1
	Non-daily smoker	870	16.8
	Former smoker	783	15.1
**Time-in-sample**	Completed one survey	2,929	56.6
	Completed two surveys	1,097	21.2
	Completed more than 2 surveys	1,149	22.2
**Survey wave of recruitment***	Wave 1 (2016)	2,633	50.9
	Wave 2 (2018)	1,569	30.3
	Wave 3 (2020)	973	18.8

Data are unweighted and unadjusted. *Survey wave of recruitment for Waves 2 and 3 is the replenishment sample only and reflects their first survey in this cohort study. Unweighted % is the sample composition for “yes”.

Supplemental Table 2. Changes in support for pictorial health warnings among current and former smokers between 2016 and 2020, overall and by smoking status

	Current daily smokers					Current non-daily smokers					Former smokers					All respondents				
**Survey Year**	**n**	**%**	**SE**	**95%**	**CI**	**n**	**%**	**SE**	**95%**	**CI**	**n**	**%**	**SE**	**95%**	**CI**	**N**	**%**	**SE**	**95%**	**CI**
2016	1829	33.5	1.58	30.5	36.7	339	40.3	4.03	32.8	48.4	389	45.9	3.36	39.4	52.5	2557	38.0	1.44	35.2	40.9
2018	1741	36.1	1.58	33.1	39.3	379	53.1	3.70	45.8	60.2	565	59.0	2.80	53.5	64.4	2685	44.7	1.38	42.0	47.4
2020	786	37.7	2.29	33.4	42.3	188	42.4	4.68	33.6	51.7	138	59.8	5.23	49.3	69.5	1112	45.0	2.19	40.7	49.3

Data are weighted. Changes in support for pictorial health warnings were assessed between 2016-2018, 2018-2020, and 2016-2020 using general estimating equations fitted with a weighted logistic regression model. The model adjusted for age, sex, income, education, race/ethnicity, smoking status, and intentions to quit smoking, and time-in-sample. SE: standard error, CI: confidence interval.

Support for pictorial health warning labels on cigarette packages in the United States among adults who currently smoke or quit smoking: Findings from the ITC US Smoking and Vaping Surveys

Supplemental Figure 1. Required Warnings for Cigarette Packages and Advertisements issued in March 2020

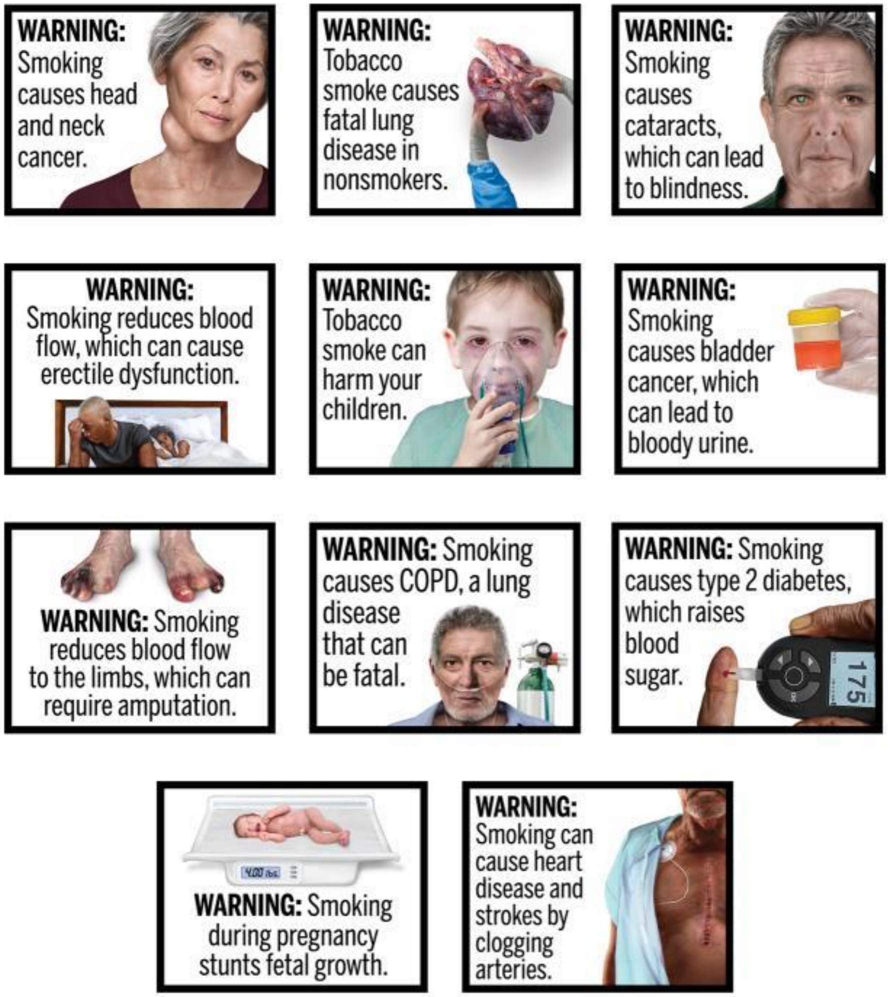

United States Food and Drug Administration. Cigarette Labeling and Health Warning Requirements. https://www.fda.gov/tobacco-products/labeling-and-warning-statements-tobacco-products/cigarette-labeling-and-health-warning-requirements#2

Supplemental Figure 2. Support for pictorial health warnings in 2020 among current and former cigarette smokers in the United States (n=1112)

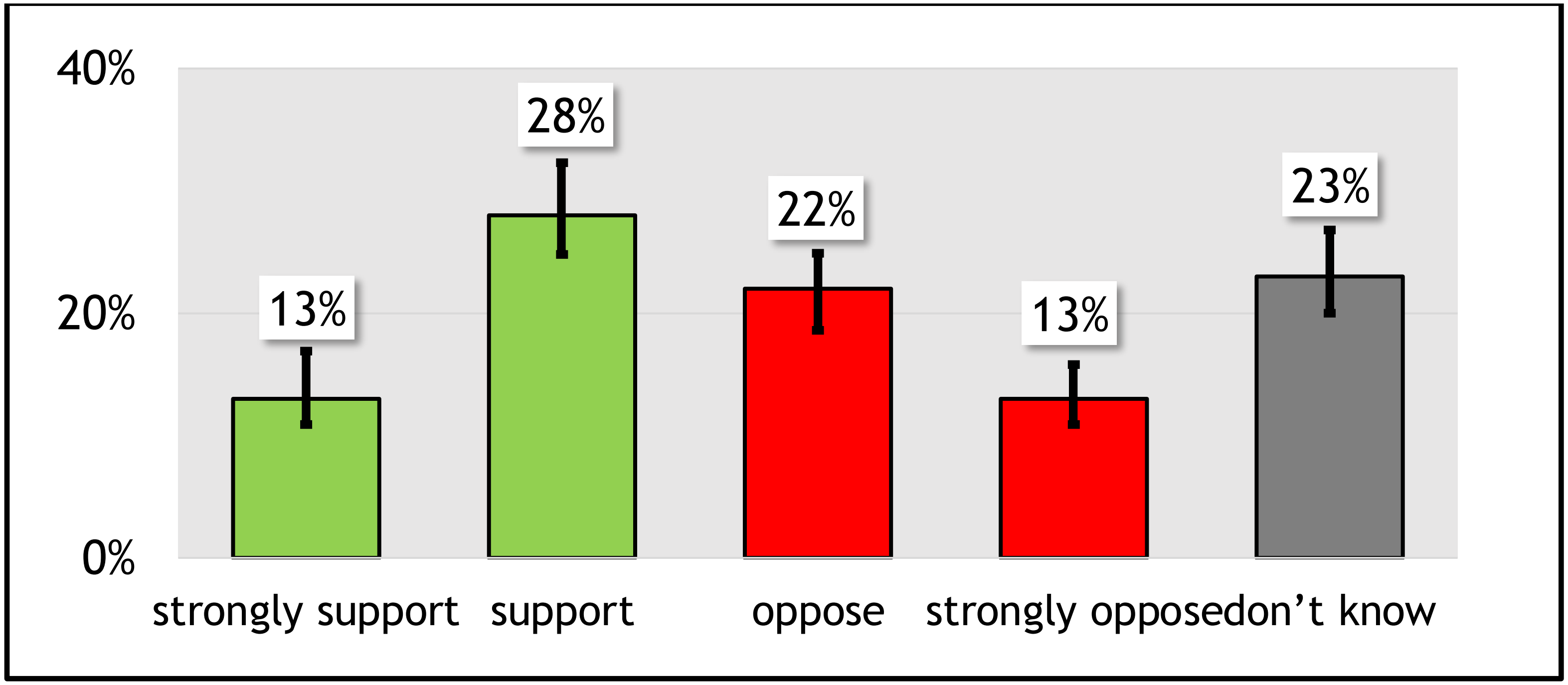

Data are weighted. A multinomial regression analysis was used to estimate the level of support for pictorial health warning labels on cigarette packages. The model adjusted for: age, sex, income, education, race/ethnicity, smoking status, time-in-sample, and plans to quit smoking.

© 2023 Gravely S. et al.
